# Polyarteritis Nodosa Following mRNA-1273 COVID-19 Vaccination: Case Study and Review of Immunological Mechanisms

**DOI:** 10.7759/cureus.33620

**Published:** 2023-01-10

**Authors:** Bahadar S Srichawla

**Affiliations:** 1 Department of Neurology, University of Massachusetts Chan Medical School, Worcester, USA

**Keywords:** coronavirus disease 2019, severe acute respiratory syndrome coronavirus 2, pfizer-biontech covid-19 vaccine related adverse events, moderna mrna adverse effects, moderna covid-19 vaccine, mrna-1273 vaccine, pfizer-biontech covid-19 vaccine, polyarteritis nodosa, sars-cov-2, covid 19

## Abstract

Numerous post-vaccine complications have been reported secondary to the COVID-19 vaccine. Many of these complications are believed to be due to a hyperactive immune system. A 59-year-old woman developed diffuse abdominal pain two days after receiving the mRNA-1273 COVID-19 vaccine (Moderna). A computerized tomography (CT) angiogram of the abdomen and pelvis revealed the presence of numerous vascular irregularities in the celiac axis, bilateral renal arteries, and inferior mesenteric artery consistent with polyarteritis nodosa (PAN), a medium-vessel vasculitis. The patient was managed with intravenous methylprednisolone 500 mg daily for three days and was then placed on oral methotrexate (MTX) 12.5 mg daily for immunosuppressive maintenance treatment. Until now, a limited number of cases of polyarteritis nodosa secondary to the COVID-19 vaccine have been reported. Major mechanisms of post-vaccine autoimmunity are molecular mimicry and autoantibody production. Although rare adverse events from COVID-19 vaccination are possible, there remains an immense benefit to vaccination in preventing COVID-19-related morbidity and mortality.

## Introduction

Coronavirus disease 2019 (COVID-19) is a pandemic that has had a significant burden on healthcare systems and patient outcomes around the world. The introduction of vaccines against severe acute respiratory syndrome coronavirus-2 (SARS-CoV-2) has allowed for improved outcomes in morbidity and mortality [1]. In the United States, the BNT162b2 mRNA (Pfizer Biotech) and mRNA-1273 (Moderna) vaccines have been approved by the Food and Drug Administration for emergency use since November 2020. Minor adverse reactions such as myalgia, fever, and aches are common. Despite this, rare severe adverse events (AEs) have been reported in the literature. Some AEs are related to overstimulation of the immune system and can include vasculitis, transverse myelitis, and neuromyelitis optica spectrum disorder among others [2]. Polyarteritis nodosa (PAN) is a systemic vasculitis of small and medium vessels that can cause tissue ischemia. Common anatomical locations include the skin, peripheral nerves, gastrointestinal (GI) tract, and kidneys among others [3]. The case of polyarteritis nodosa (PAN) reported here occurred approximately two days after receiving the second dose of the original mRNA-1273 vaccine.

## Case presentation

**Figure 1:**
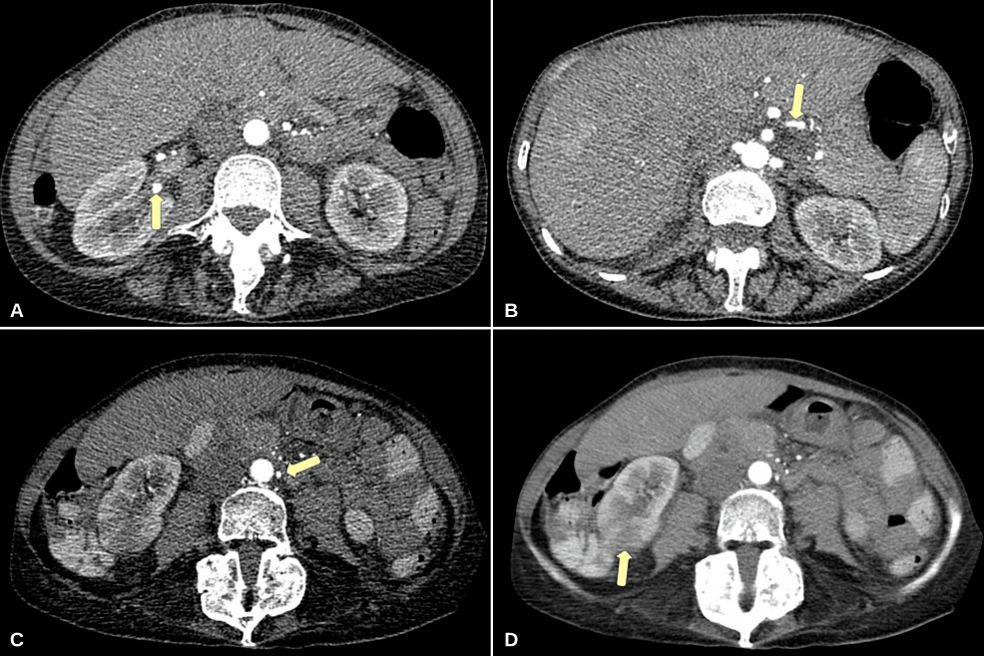
Initial computerized tomography (CT) angiography of the abdomen and pelvis (A) Irregularity of the distal portion of the right renal artery with multifocal aneurysmal dilatation. (B) Slight narrowing at the proximal aspect of the splenic artery. (C) Irregularity and dilatation of the proximal aspect of the inferior mesenteric artery. (D) Heterogenous hypo-enhancement of the posterior aspect of the right kidney.

A healthy 59-year-old woman presented to the emergency department with a two-day history of progressively worsening abdominal pain, melena, and postprandial nausea. The patient had no significant chronic medical history and no recent infections. She was recently vaccinated with the second dose of the mRNA-1273 vaccine against SARS-CoV-2 approximately two days before her presentation. Physical examination was significant for diffuse tenderness to palpation of the abdomen with rebound tenderness of the right upper quadrant. Laboratory values were significant for a mild leukocytosis with a WBC count of 12,550 cells/mL (4,500-11,000), and an elevated erythrocyte sedimentation rate (ESR) of 91 mm/h (0-21). Serology for both hepatitis B and C was negative. Serum antibody testing was significant for a positive antinuclear antibody (ANA) with a level of titer of 1:160. Both myeloperoxidase-anti neutrophil cytoplasmic antibody (ANCA), and proteinase-3 antibody screening were negative. Urine analysis showed no gross blood and 3-5 red blood cells/high power field with no WBCs. The patient tested negative for SARS-CoV-2 via polymerase chain reaction (PCR). A computerized tomography (CT) scan of the abdomen showed numerous vascular irregularities in the celiac axis, bilateral renal arteries, and inferior mesenteric artery consistent with vasculitis. There was significant stenosis of the distal branch of the right renal artery resulting in renal parenchyma ischemia with heterogenous enhancement (Figure 1).

**Figure 2:**
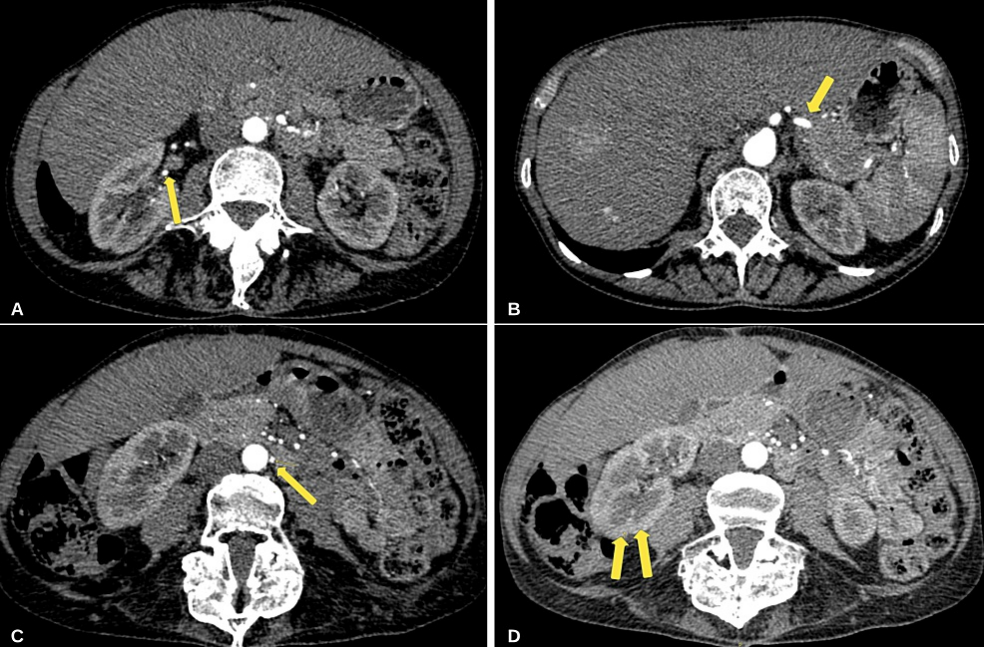
Repeat CT abdomen and pelvis angiography two months after discharge. (A-C) Resolution of prior vascular irregularities of the left renal, splenic, and inferior mesenteric arteries. (D) Resolution of renal parenchymal hypo-enhancement.

The patient was subsequently diagnosed with polyarteritis nodosa, a medium-vessel vasculitis, and was started on intravenous methylprednisolone 500 mg for three days with a one-week tapering dose. Her symptoms had significantly improved, and she was started on methotrexate (MTX) 12.5 mg daily for remission maintenance immunosuppression. At a three-month follow-up, the patient had a subsequent CT angiogram of the abdomen which showed resolution of the previous vascular irregularities and renal ischemia (Figure 2). At a one-year follow-up appointment, the patient remains in remission with the same MTX therapy.

## Discussion

PAN is a medium-sized vessel vasculitis and is often seen in middle-aged women such as the one reported here. The worldwide incidence of PAN is decreasing which is largely attributed to the hepatitis B vaccine (HBV). The clinical presentation of PAN often involves fever, weight loss, malaise, and joint pain. In patients with gastrointestinal involvement; abdominal pain, melena, and vomiting are often reported. Other organ systems involved include the coronary arteries (~35%), kidneys (~60%), and skin (~40%) [4]. Moderate and severe PAN is defined as those with systemic organ involvement outside of the integumentary system. The respiratory system is often spared in PAN. Laboratory results may show an elevated ESR, C-reactive protein (CRP), leukocytosis, and anemia [5]. Similarly, in our case, we observed the elevation in these inflammatory markers.

PAN involving the abdomen includes inflammation manifested as stenosis and aneurysmal dilatation of various medium-sized vessels. Renal artery involvement is the most prevalent. A retrospective review of seven patients with PAN involving the abdomen reported five cases of renal artery aneurysms and six cases with involvement of the mesenteric artery [6]. Systemic vasculitides are stratified by small, medium, or large vessel involvement. The onset of inflammation in medium-sized vasculitis such as PAN is more acute than that of large-vessel vasculitis [7]. Similarly, in the presented case the onset of abdominal pain and medium-vessel vasculitis was observed on imaging a few days after COVID-19 vaccination.

For non-HBV-related PAN, the management is similar to other forms of vasculitis. First-line management for moderate and severe PAN includes high-dose glucocorticoids and/or cyclophosphamide. In cases of HBV-associated PAN antiviral therapy or plasmapheresis can be administered. Maintenance immunosuppression therapy can include azathioprine and MTX. Immunosuppressive therapy is often used for a total duration of 18 months [8]. However, in cases of mild PAN long-term immunosuppressive therapy may not be needed. Until now, only a few cases of PAN have been reported secondary to COVID-19 vaccination. Su et al. reported on a 52-year-old woman who developed cutaneous PAN approximately one month after vaccination with the ChAdOx1 nCoV-19 vaccine. The patient was managed with daily methylprednisolone and monthly oral methotrexate [9]. Ohkubo et al. reported a 61-year-old man diagnosed with PAN and epididymitis following the BNT162b2 vaccine. The patient was treated with prednisolone and antibiotics [10]. Prior analysis of the Vaccine Adverse Event Reporting System (VAERS) has described 10 cases of possible PAN from the HBV vaccine [11].

**Figure 3:**
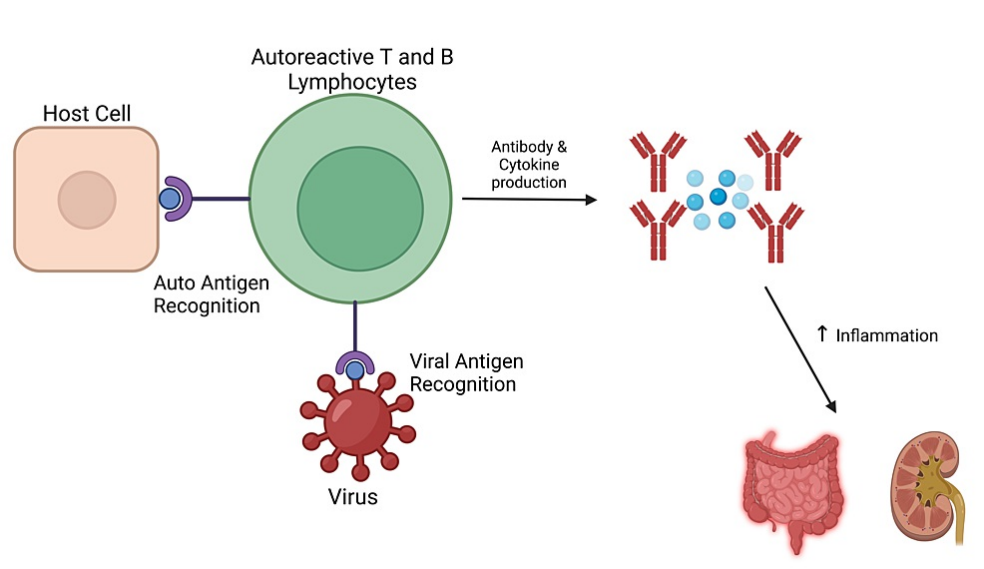
Molecular mimicry hypothesis This figure was created using BioRender [17]

The COVID-19 mRNA vaccine may lead to an acute increase in inflammatory markers after vaccination, particularly after the second dose. The mRNA has been shown to bind pattern recognition receptors prior to translation and lead to the activation of multiple pro-inflammatory cascades. Up-regulation of these immunological mechanisms may lead to the observed autoimmune conditions [12]. Molecular mimicry (MM) is one proposed mechanism of vaccine-associated autoimmunity. MM occurs due to a similarity in pathogenic elements in a vaccine and specific human proteins [13]. Figure 3 provides a visual depiction of cellular mechanisms observed in molecular mimicry. Autoreactive T-lymphocytes inappropriately react to self-derived peptides leading to auto-antibody and cytokine production. Similarly, this 'cross-reactivity' mechanism may occur secondary to vaccine-derived peptides. Other mechanisms of autoimmunity include B-cell immortalization, bystander activation, epitope spreading, and thymus dysregulation [14]. Next-generation sequencing technology has shown to be useful in pathogen recognition and may also have an important role in diagnosing autoimmune conditions [15]. Possible applications include microRNA (miRNA) expression patterns and HLA gene polymorphisms [16].

A descriptive cohort analysis of 3.9 million individuals vaccinated with an mRNA-based (BNT162b2) and a non-mRNA-based (CoronaVac) vaccine revealed a similar incidence of autoimmune conditions compared to nonvaccinated individuals [18]. Larger international multicenter longitudinal studies are needed to further elaborate these findings. Further research is required on the reactogenicity of mRNA-based COVID-19 vaccines in those with a history of autoimmune conditions. Consideration for immunosuppressive therapy in those receiving mRNA- based vaccines with known autoimmunity can be given [19].

## Conclusions

The case of a 59-year-old woman who was diagnosed with polyarteritis nodosa two days after mRNA-1273 COVID-19 vaccination is reported. CT angiography of the abdomen and pelvis revealed multifocal aneurysmal dilatation of the renal artery, stenosis of the splenic artery, and dilatation of the proximal inferior mesenteric artery. Management included high-dose intravenous methylprednisolone for three days and methotrexate for maintenance immunosuppression. Few cases of PAN secondary to COVID-19 vaccination have been reported thus far. Immunologic mechanisms associated with post-vaccine autoimmunity include cytokine storm, autoantibody production, and molecular mimicry. Although rare AEs secondary to the COVID-19 vaccine are possible, there remains an immense benefit to COVID-19-related morbidity and mortality.
